# Vitamin D deficiency, fatigue, and persistent cough as independent predictors of depressive symptoms in sarcoidosis patients

**DOI:** 10.5937/jomb0-54015

**Published:** 2025-06-13

**Authors:** Branislav S. Gvozdenovic, Violeta Mihailovic-Vucinic, Mira Vukovic, Maja Omcikus, Jelena Cvejic, Slobodan Belic, Jelena Jankovic, Natasa Đurđevic, Mihailo Stjepanovic

**Affiliations:** 1 PPD Serbia, Part of Thermo Fisher Scientific, Pharmacovigilance Department, Belgrade; 2 MediGroup General Hospital, Belgrade, Serbia; 3 University of Belgrade, School of Medicine, Belgrade; 4 Health Center Valjevo, QA Department; 5 University of Belgrade, School of Medicine, Belgrade, Serbia; 6 University Clinical Center of Serbia, Clinic for Pulmonary Diseases, Belgrade

**Keywords:** sarcoidosis, depressive symptoms, vitamin D deficiency, fatigue, cough, multivariate analysis, sarkoidoza, simptomi depresije, nedostatak vitamina D, zamor, kašalj, multivarijantna analiza

## Abstract

**Background:**

Depressive symptoms are frequent in sarcoidosis. We assessed the impact of sarcoidosis symptoms, pulmonary function, fatigue, radiographic findings, comorbidities, treatment, and serum levels of 25-hydroxyvitamin D (25(OH)D) on depressive symptoms in sarcoidosis patients.

**Methods:**

In a cross-sectional study, we measured depressive symptoms using the Center for Epidemiologic Studies - Depression Scale (CES-D) and fatigue using the Fatigue Assessment Scale (FAS). Presence of depressive symptoms was defined with CES-D scores ≥16 and ≥20, respectively. Fatigue was defined as having an FAS score of ≥22.

**Results:**

A total of 400 patients were included in the study. CES-D score ≥16 had 128 patients, while CES-D score ≥20 had 86 patients. In a multivariate binomial logistic regression model, the following independent predictors of CES-D score ≥16 were identified: female gender (odds ratio, OR 1.983), chronic sarcoidosis (OR 2.311), serum levels of 25(OH)D ≤20 ng/mL (OR 2.326), persistent dry cough (OR 2.173), FAS Scores ≥22 (OR 9.243), and chest X-ray stage 3 (8.851). Five variables were independent predictors of CES-D score ≥20: diplopia (OR 4.411), FEV1 <80% predicted associated with FVC <80% predicted (OR 2.311), serum levels of 25(OH)D ≤20 ng/mL (OR 2.278), persistent dry cough (OR 3.001), and FAS Scores ≥22 (OR 7.611).

**Conclusions:**

Measuring the contribution of low serum 25-hydroxyvitamin D and the impact of persistent dry cough on depressive symptoms in patients with sarcoidosis may be crucial in deciding whether to use vitamin D3 alone or with antitussive therapy before the psychiatric diagnosis of depression with antidepressant therapy initiation.

## Abstract (sr)

**Uvod:**

Simptomi depresije su česti kod sarkoidoze. Ispitivali smo uticaj simptoma sarkoidoze, plućne funkcije, zamora, radiografskih nalaza, komorbiditeta, lečenja i nivoa 25-hidrok si vitamina D (25(OH)D) u serumu na simptome depresije kod pacijenata sa sarkoidozom.

**Metode:**

U okviru studije preseka, simptomi depresije su mereni pomoću Skale za procenu depresije Centra za epidemiološke studije (CES-D), a zamor pomoću Skale za procenu zamora (FAS). Prisustvo simptoma depresije definisano je CES-D skorovima ≥16 i ≥20, dok je zamor definisan FAS skorom od ≥22.

**Rezultati:**

Ukupno 400 pacijenata je uključeno u studiju. CES-D skor ≥16 je imalo 128 pacijenata, dok je CES-D skor ≥20 imalo 86 pacijenata. U multivarijantnom binomi jalnom logističkom regresionom modelu identifikovani su sledeći nezavisni prediktori za CES-D skor ≥16: ženski pol (OR 1,983), hronična sarkoidoza (OR 2,311), serumski nivo 25(OH)D ≤20 ng/mL (OR 2,326), uporan suvi kašalj (OR 2,173), FAS skor ≥22 (OR 9,243) i stadijum 3 na rentgenu grudnog koša (OR 8,851). Pet varijabli su bili nezavisni prediktori za CES-D skor ≥20: diplopija (OR 4,411), FEV1 <80% od predvi|enog udružen sa FVC <80% od pred vi|enog (OR 2,311), serumski nivo 25(OH)D ≤20 ng/mL (OR 2,278), uporan suvi kašalj (OR 3,001) i FAS skor ≥22 (OR 7,611).

**Zaključak:**

Merenje doprinosa niskog serumskog nivoa 25-hidroksivitamina D i uticaja upornog suvog kašlja na simp tome depresije kod pacijenata sa sarkoidozom može biti ključno u odlučivanju da li koristiti vitamin D3 samostalno ili u kombinaciji sa antitusivnom terapijom pre postavljanja psihijatrijske dijagnoze depresije i započinjanja terapije antidepresivima.

## Introduction

Sarcoidosis is a multisystem inflammatory granulomatous disorder of unknown aetiology, primarily affecting the lungs. Sarcoidosis patients have a reduced quality of life [1] and are at an increased risk for several comorbidities [2]. A significant number of sarcoidosis patients have depression [3], especially when compared with the general population [4]. The influence of different subjective and objective disease characteristics in sarcoidosis patients on their symptoms of depression has been addressed in several studies [3] [4]. Sikjær et al. [5] demonstrated that sarcoidosis patients had a higher risk of anxiety and/or depression compared with matched comparators during 18 years of follow-up. De Kleijn et al. [6] reported the combination of fatigue and high levels of depressive symptoms (DS) in 43%–46% of sarcoidosis patients. Vitamin D deficiency in sarcoidosis is associated with the course of chronic disease, significant impairment of lung function, fatigue, and depression [7]. However, there is a lack of studies with multivariateanalysis where the partial or combined contributions of vitamin D deficiency, fatigue, and other previously mentioned predictors of DS in sarcoidosis patients were jointly measured. Also, it has not been precisely established whether and to what extent they are mutually independent predictors of depression.

This study assessed the independent impacts of sarcoidosis symptoms, pulmonary function, fatigue, radiographic findings, comorbidities, disease treatment, and vitamin D deficiency on DS in sarcoidosis patients.

## Materials and methods

This study was conducted at the Clinic for Pulmonology of the University Clinical Center of Serbia in Belgrade from July 2021 to June 2023. We examined consecutive adult patients with biopsyproven pulmonary sarcoidosis. All patients were ≥18 years old, and they had not been treated for depression. All patients performed pulmonary function testing, measured serum levels of 25-hydroxyvitamin D (25(OH)D) and serum calcium, and completed questionnaires for DS and fatigue. Detailed anamnestic data were obtained regarding the course of the disease (acute – up to 2 years duration vs chronic – 2 or more years duration) and its treatment, together with comorbidities and accompanying symptoms.

This study was approved by the local Ethics Committee of the Clinic for Pulmonary Diseases, University Clinical Center of Serbia, Belgrade.

According to Holick et al. [8], we defined vitamin D deficiency as a serum level of 25(OH)D below 20 ng/mL. The normal serum calcium range per our laboratory is 2.25–2.55 mmol/L.

Depressive symptoms were measured by the Center for Epidemiologic Studies – Depression Scale (CES-D) [9]. It was initially developed to measure the current level of the respondent’s DS in epidemiological studies in the general population and primary care, and it has also been extensively used in other chronic conditions [10] [11] and even as a standalone depression diagnostic measure [12]. It has also been used in sarcoidosis patients [13]. The scale contains 20 items related to symptoms occurring the week before the interview, with response options from 0 to 3 that refer to the frequency of the symptoms. The CES-D score ranges from 0 (best possible) to 60 (worst). We applied the cut-off points of both 16 – typically recommended for screening DS [14], and 20 – which eliminates responders with false-positive DS while achieving a better relationship between sensitivity and specificity of the CES-D [15].

Fatigue was assessed by the standardized Fatigue Assessment Scale (FAS) [16]. It is a 10-item self-reported fatigue questionnaire. Answers are offered on a 5-point Likert scale (1 never to 5 always). Total scores on the FAS can range from 10 to 50, with higher scores indicating greater fatigue. FAS total score ≥22 meets the definition of fatigue. The psychometric properties of the FAS are good in sarcoidosis patients [17] [18] [19]. The Serbian language version has undergone validation in sarcoidosis [20] [21]. De Vries et al. demonstrated good test-retest reliability of the FAS with a correlation of 0.89 (p<001) for a oneweek interval [22]. In the study of Michielsen et al. [16], the internal consistency of the FAS was 0.90 Exploratory factor analysis of the FAS items showed a unique factor supported by the scree plot. The factor loadings varied from 0.82 to 0.55. The factor explained 53% of the variance.

On the same day, subjects completed the questionnaires with laboratory sampling and performed spirometry and the transfer factor of the lung for carbon monoxide (DLCO). Spirometry parameters included pre-bronchodilator forced expiratory vital capacity (FVC), forced expiratory volume in one second (FEV_1_), and the ratio FEV_1_/FVC. It was measured with a pneumotachograph (Masterlab, Jaeger, Wurzburg, Germany). For DLCO measuring, the single- breath method (Masterlab, Jaeger, Wurzburg, Germany) was applied. The European Respiratory Society criteria for lung function impairments were used [23].

### Statistical analysis

Data sets for continuous numerical variables are described by mean and standard deviation, whileattributive variables are presented by frequency and percentage. Univariate analysis of differences between groups for nominal variables was performed using Pearson’s chi-square test or Fisher’s exact probability test. Student t-test was used for univariate analysis of differences in continuous variables. The method of binary logistic regression with stepwise selection of variables was used for multivariate analysis of risk factors for increased DS. All variables with p <0.2 in univariate analyses were considered for inclusion in the final multivariate regression model.

Validation of the logistic regression model included the assessment of its goodness-of-fit measure and its accuracy. The best-fitting model was created by estimating the Nagelkerke R2 parameter. The accuracy of the logistic regression model was assessed by discrimination analysis and its adequacy. Discriminant analysis (c-statistic or area under the curve) was performed to demonstrate how well the model could distinguish patients who have DS (CES-D score ≥16 or CES-D score ≥20) from those who do not have them. The analysis of the adequacy of logistic models and the evaluation of the retention of variables (or their interactions) was performed using the Hosmer-Lemeshow method. A variance inflation factor (VIF) of less than 3 will determine the absence of major multicollinearity in the logistic regression model. After estimating the best model and obtaining the appropriate regression equation, that model was used to develop the Depressive Symptoms Risk Index for Sarcoidosis Patients (DSRISP). The number of points assigned to each variable represented the regression coefficient divided by 0.5, after which the value thus obtained was rounded to the nearest whole number. The scores for each risk factor were then summed in the total number of points (score) for each patient. A »Receiver Operating Characteristics« (ROC) procedure was performed for the DSRISP score discrimination analysis. Criterion value, sensitivity, specificity, positive predictive value, and negative predictive value were obtained based on assessing the maximum Youden index. The level of estimated statistical significance was 0.05. Data processing was performed using the IBM SPSS Statistics 20 (NY).

## Results

A total of 400 sarcoidosis patients were included in the study, with an average age of 50.52±11.00 years, and 72.8% of patients were women. Other patients’ characteristics are shown in Table 1. Depressive symptoms had 32%, according to the CES-D score ≥16, and 21.5%, when using a CES-D cut-off score ≥20.

**Table 1:** Description of sarcoidosis patients’ characteristics in the total study population (N=400). SD – Standard Deviation; f – Frequency; FEV_1_ – Forced Expiratory Volume in one second; FVC – Forced Expiratory Vital Capacity; DLCO – transfer factor of the lung for carbon monoxide; FAS – Fatigue Assessment Scale; 25(OH)D ‒ 25-hydroxyvitamin D; CES-D – Center for Epidemiologic Studies Depression Scale.

		Mean ± SD
Age (years)		50.52±11.00
Disease duration (years)		6.08±6.14
FVC (L)		3.74±1.08
FEV_1_ (L)		2.96±0.93
FVC (% predicted)		107.33±16.43
FEV_1_ (% predicted)		100.79±18.64
FEV_1_ / FVC		80.29±9.30
DLCO (L)		6.85±1.99
DLCO (% predicted)		87.03±15.49
Serum level of 25(OH)D (ng/mL)		14.23±10.18
Serum calcium (mmol/L)		2.31±0.62
FAS Scores		21.91±8.30
CES-D Scores		14.38±8.75
		f (%)
Gender	Male	109 (27.3)
	Female	291 (72.8)
Extrapulmonary<br>sarcoidosis	No	99 (24.8)
	Yes	301 (75.3)
Course	Chronic	236 (59.0)
	Acute	164 (41.0)
Treatment	Prednisone	116 (29.0)
	Methotrexate	244 (61.0)
	Chloroquine	27 (6.8)
	Prednisone + Methotrexate	13 (3.3)
The most<br>common<br>comorbidities	Arterial hypertension	278 (69.5)
	Arrhythmia	7 (1.8)
	Angina pectoris	7 (1.8)
	Diabetes mellitus	360 (90.0)
	Disease of the<br>thyroid gland	10 (2.5)
	Anemia	5 (1.3)
Symptoms	Persistent dry cough	340 (85.0)
	Shortness of breath	78 (19.5)
	Fatigue	276 (69.0)
	Chest pain	63 (15.8)
	Bone pains	229 (57.3)
	Joint swelling	75 (18.8)
	Skin changes	38 (9.5)
	Diplopia	14 (3.5)

Univariate analysis showed that patients with CES-D scores ≥16 compared to those with CES-D scores <16 were older, had longer disease duration, higher FAS scores, lower serum 25(OH)D and lower DLCO. Also, patients with CES-D scores ≥20 compared to those with CES-D scores <20 were older, had longer disease duration, had higher FAS scores, and had lower DLCO (Table 2).

**Table 2:** Univariate assessment of differences in continuous characteristics of sarcoidosis patients between No/Yes the CES-D score ≥16 groups and between No/Yes the CES-D score ≥20 groups.

	CES-D Scores ≥16				CES-D Scores ≥20			
		N	Mean ± SD	p(t)		N	Mean ± SD	p(t)
Age (years)	No	272	48.86±11.03	0.000<br>(-4.498)	No	314	49.57±10.97	0.001<br>(-3.454)
	Yes	128	54.05±10.11		Yes	86	54.01±10.46	
Disease<br>duration (years)	No	272	5.48±5.77	0.004<br>(-2.862)	No	314	5.61±5.76	0.011<br>(-2.600)
	Yes	128	7.35±6.73		Yes	86	7.79±7.16	
Serum 25(OH)D<br>(ng/mL)	No	272	15.23±10.43	0.003<br>(3.001)	No	314	14.72±10.20	0.064<br>(1.860)
	Yes	128	12.11±9.32		Yes	86	12.43±9.96	
Serum Calcium<br>(mmol/L)	No	272	2.31±0.64	0.990<br>(0.013)	No	314	2.31±0.60	0.763<br>(-0.302)
	Yes	128	2.31±0.56		Yes	86	2.33±0.70	
FAS Scores	No	272	18.97±6.65	0.000<br>(-11.271)	No	314	19.92±7.04	0.000<br>(-9.224)
	Yes	128	28.16±8.02		Yes	86	29.17±8.54	
FVC<br>(% predicted)	No	272	107.96±16.21	0.262<br>(1.124)	No	314	108.15±15.89	0.057<br>(1.905)
	Yes	128	105.98±16.89		Yes	86	104.35±18.07	
FEV_1_<br>(% predicted)	No	272	101.92±17.79	0.077<br>(1.772)	No	314	101.68±18.06	0.067<br>(1.834)
	Yes	128	98.39±20.20		Yes	86	97.53±20.43	
FEV_1_/FVC	No	272	80.69±8.79	0.209<br>(1.259)	No	314	80.41±9.32	0.603<br>(0.520)
	Yes	128	79.43±10.29		Yes	86	79.82±9.26	
DLCO<br>(% predicted)	No	272	88.71±14.30	0.005<br>(2.829)	No	314	80.12±14.71	0.014<br>(2.459)
	Yes	128	83.65±17.23		Yes	86	83.05±17.60	

Univariate analyses of differences according to categorical characteristics (Table 3) showed that the group with CES-D score ≥16 compared to the group with CES-D score <16 was more often represented by: women, aged ≥65 years, extrapulmonary sarcoidosis, chronic disease course, treatment with a combination of prednisone + methotrexate, chest X-ray (CXR) stage 3, FAS Scores ≥22, FEV_1_/FVC ≥0.70 and serum 25(OH)D <20 ng/mL. In addition, patients with CES-D score ≥16 had a higher prevalence of the following symptoms and comorbidities: arterial hypertension, persistent dry cough, fatigue, chest pain, shortness of breath, bone pains, skin changes and joint swelling. Similar results were seen between groups with CES-D score thresholds ≥ or <20, except for age ≥65 years, treatment, CXR stage, and FEV_1_/FVC <0.70 (Table 3). Additionally, the group with a CES-D score ≥20 had a higher prevalence of combined occurrence of FVC <80% predicted and FEV_1_ <80% predicted, as well as more frequent diplopia.

**Table 3:** Univariate assessment of differences in nominal characteristics of sarcoidosis patients between No/Yes (272 patients/128 patients) the CES-D score ≥16 groups and between No/Yes (314 patients/86 patients) the CES-D score ≥20 groups. χ^2^ – Hi square value; FAS – Fatigue Assessment Scale; 25(OH)D – 25-hydroxyvitamin D; CES-D – Center for Epidemiologic Studies Depression Scale; CXR – Chest X-Ray.

		CES-D ≥16		p(x^2^)	CES-D ≥20		p(x^2^)
		No (%)	Yes (%)		No (%)	Yes (%)	
Gender	Male	32.7	15.6	0.000 (12.832)	29.9	17.4	0.021 (5.316)
	Female	67.3	84.4		70.1	82.6	
Age ≥65 years	No	93.0	86.7	0.040 (4.213)	92.4	86.0	0.070 (3.282)
	Yes	7.0	13.3		7.6	14.0	
Extrapulmonary<br>sarcoidosis	No	29.4	14.8	0.002 (9.918)	27.4	15.1	0.019 (5.459)
	Yes	70.6	85.2		72.6	84.9	
Course	Chronic	53.3	71.1	0.001 (11.381)	55.7	70.9	0.011 (6.446)
	Acute	46.7	28.9		44.3	29.1	
Treatment	Prednisone	33.1	20.3	0.003 (13.951)	30.3	24.4	0.139 (5.459)
	Methotrexate	59.2	64.8		60.8	61.6	
	Chloroquine	6.2	7.8		6.7	7.0	
	Prednisone +<br>Methotrexate	1.5	7.0		2.2	7.0	
Corticosteroid<br>therapy	No	65.4	72.6	0.150 (2.274)	67.5	68.6	0.848 (0.037)
	Yes	34.6	27.4		32.5	31.4	
CXR Stage	0	35.7	24.2	0.000 (20.290)	32.5	30.2	0.855 (0.778)
	1	29.0	31.2		29.6	30.2	
	2	34.6	35.9		35.0	34.9	
	3	0.7	8.6		2.9	4.7	
Anemia	No	98.9	98.4	0.657	98.7	98.8	1.000
	Yes	1.1	1.6		1.3	1.2	
Angina pectoris	No	98.5	96.9	0.274	98.4	96.5	0.337
	Yes	1.5	3.1		1.6	3.5	
Arrhythmia	No	98.9	96.9	0.217	98.4	97.7	0.646
	Yes	1.1	3.1		1.6	2.3	
Disease of the<br>thyroid gland	No	98.2	96.1	0.301	97.8	96.5	0.454
	Yes	1.8	3.9		2.2	3.5	
Arterial<br>hypertension	No	73.5	60.9	0.011 (6.511)	72.3	59.3	0.020 (5.375)
	Yes	26.5	39.1		27.7	40.7	
Diabetes mellitus	No	91.2	87.5	0.253 (1.307)	90.8	87.2	0.330 (0.948)
	Yes	8.8	12.5		9.2	12.8	
FAS Scores ≥22	No	69.9	18.8	0.000 (91.370)	63.7	16.3	0.000 (61.009)
	Yes	30.1	81.2		36.3	83.7	
Persistent dry<br>cough	No	90.1	74.2	0.000 (17.160)	89.2	69.8	0.000 (19.937)
	Yes	9.9	25.8		10.8	30.2	
Fatigue	No	40.8	10.2	0.000 (38.233)	36.6	10.5	0.000 (21.597)
	Yes	59.2	89.8		63.4	89.5	
Chest pain	No	92.3	67.2	0.000 (41.299)	88.9	67.4	0.000 (23.325)
	Yes	7.7	32.8		11.1	32.6	
Shortness of<br>breath	No	90.4	59.4	0.000 (53.514)	86.9	57.0	0.000 (38.618)
	Yes	9.6	40.6		13.1	43.0	
Bone pains	No	54.8	17.2	0.000 (50.257)	50.6	14.0	0.000 (37.119)
	Yes	45.2	82.8		49.4	86.0	
Skin changes	No	95.2	80.5	0.000 (22.031)	92.7	82.6	0.005 (8.037)
	Yes	14.8	19.5		7.3	17.4	
Diplopia	No	97.8	93.8	0.075	98.1	90.7	0.001 (10.920)
	Yes	2.2	6.2		1.9	9.3	
Joint swelling	No	88.6	65.6	0.000 (30.166)	86.6	61.6	0.000 (27.688)
	Yes	11.4	4.4		13.4	38.4	
Serum Calcium >2.55<br>mmol/L	No	98.9	98.7	1.000	99.0	98.0	0.491
	Yes	1.1	1.3		1.0	2.0	
Serum 25(OH)D <20<br>ng/mL	No	25.7	12.5	0.003 (9.034)	24.2	11.6	0.012 (6.326)
	Yes	74.3	87.5		75.8	88.4	
FVC predicted < 80%	No	94.9	93.0	0.492	95.5	89.5	0.062
	Yes	5.1	7		4.5	10.5	
FEV_1_ predicted < 80%	No	89.3	82.8	0.068 (3.332)	89.2	80.2	0.028 (7.850)
	Yes	10.7	17.2		10.8	19.8	
FEV_1_ / FVC < 70	No	8.5	16.4	0.018 (5.620)	9.6	16.3	0.077 (3.119)
	Yes	91.5	83.6		90.4	83.7	
FVC predicted < 80% and<br>FEV_1_ predicted < 80%	No	96.0	93.0	0.222	96.5	89.5	0.021
	Yes	4.0	7.0		3.5	10.5	

The first multivariate binomial logistic regression model yielded six independent predictors for the CES-D score ≥16 (Table 4): female gender (odds ratio, OR 1.983), chronic course of sarcoidosis (OR2.311), serum level of 25(OH)D <20 ng/mL (OR 2.326), persistent dry cough (OR 2.173), FAS scores ≥22 (OR 9.243), and CXR stage 3 (OR 8.851).

**Table 4:** Parameters of the multivariate binomial logistic regression model in predicting CES-D score ≥16 and assigning corresponding points for the construction of the Depression Symptoms Risk Index 1 for Sarcoidosis Patients. B – Regression coefficients; SE – Standard Error; CI – Confidence Interval; FAS – Fatigue Assessment Scale; 25(OH)D – 25-hydroxyvitamin D; CES-D – Center for Epidemiologic Studies Depression Scale; CXR – Chest X-Ray.

	B	SE	Wald	df	p	Odds<br>ratio	95% CI for the odds ratio		Points
							Lower limit	Upper limit	
Course (Acute) – *Reference*	/	/	/	/	/	/	/	/	0
Course (Chronic)	0.838	0.285	8.640	1	0.003	2.311	1.322	4.040	+2
Serum 25(OH)D ≤20 ng/mL<br>(No)	/	/	/	/	/	/	/	/	0
Serum 25(OH)D ≤20 ng/mL<br>(Yes)	0.844	0.347	5.912	1	0.015	2.326	1.178	4.594	+2
Persistent dry cough (No) –<br>*Reference*	/	/	/	/	/	/	/	/	0
Persistent dry cough (Yes)	0.776	0.364	4.558	1	0.033	2.173	1.066	4.432	+2
Gender (Male) – *Reference*	/	/	/	/	/	/	/	/	0
Gender (Female)	0.685	0.337	4.140	1	0.042	1.983	1.025	3.836	+1
FAS Scores ≥22 (No) –<br>*Reference*	/	/	/	/	/	/	/	/	0
FAS Scores ≥22 (Yes)	2.224	0.287	60.028	1	0.000	9.243	5.266	16.222	+4
CXR Stage	/	/	10.277	3	0.016	/	/	/	/
CXR Stage (0) – *Reference*	/	/	/	/	/	/	/	/	0
CXR Stage (1)	0.513	0.340	2.280	1	0.131	1.671	0.858	3.254	0
CXR Stage (2)	-0.227	0.335	0.460	1	0.498	0.797	0.413	1.537	0
CXR Stage (3)	2.181	0.934	5.456	1	0.020	8.851	1.420	55.164	+4
Constant	-4.026	0.540	55.508	1	0.000	0.018			

Based on the regression coefficients (Table 4), all of which were first divided by 0.5 and then rounded to the nearest whole number, points were assigned to form the DSRISP 1 score in the following way: course (Chronic) +2 points, serum level of 25(OH)D <20 ng/mL (Yes) +2 points, persistent dry cough (Yes) +2 points, gender (Female) +1 point, FAS Scores ≥22 (Yes) +4 points and CXR Stage 3 (Yes) +4 points. The DSRISP 1 score is obtained by summing all associated points for the indicated risk of the CES-D score ≥16. Its score values from 0 (the lowest risk for the CES-D score ≥16) to 15 (the highest risk).

Omnibus models testing showed that the model coefficients at each subsequent step during the stepwise process were significant and that the estimation of parameters was terminated at iteration number 5 because parameter estimates changed by less than 0.001. The model parameters (R^2^ and -2 log likelihood), by steps with added variables in each subsequent iteration (from step 1 to step 6), are shown in the following order: 1) (with constant) FAS Scores ≥22 (R^2^=0.299, -2 log likelihood=405.465); 2) Chronic course of sarcoidosis (R^2^=0.325, -2 log likelihood=395.965); 3) CXR (R^2^=0.365, -2 log likelihood=380.468); 4) Serum 25(OH)D ≤20 ng/mL(R^2^=0.378, -2 log likelihood=375.686); 5) Persistent dry cough (R^2^=0.390, -2 log likelihood=370.795); 6) Gender (R^2^=0.401, -2 log likelihood=366.470).

This first model (Table 4) showed good construct validity (Nagelkerke R2=0.401), excellent adequacy by the Hosmer-Lemesh test (Chi-square=4.039; p=0.854), and good discriminant characteristics (c-statistic = 0.780 with 95% confidence interval (CI) from 0.737 to 0.820, p<0.0001). The ROC procedure assessed the classification characteristics of the DSRISP 1 score, with its cut-off points >5 (with a 95% CI from 4 to 5), which were evaluated as a criterion value for classifying the patient into the group with CES-D ≥16. Other ROC parameters of the DSRISP 1 score with a 95% CI were: sensitivity = 80.5% (72.5%–86.9%), specificity = 77.0% (65.2%–76.3%), positive predictive value = 56.6% (49.1%–63.9%) and negative predictive value = 88.5% (83.5–92.4%).

The second multivariate binomial logistic regression model yielded five independent predictors for a CES-D score ≥20 (Table V), with the corresponding number of points assigned to the Depressive Symptom Risk Index 2 (DSRISP 2) construct. Their score values range from 0 (the lowest risk for the CES-D score ≥20) to 13 (the highest risk).

The high odds ratio (Table 4 and Table 5) associated with FAS Scores ≥22 with the leading partial R2 relative to the partial contributions to the R2 of the other predictors in our logistic regression models suggests fatigue’s critical role in DS.

**Table 5:** Parameters of the multivariate binomial logistic regression model in predicting CES-D score ≥20 and assigning corresponding points for the construction of the Depression Symptoms Risk Index 2 for Sarcoidosis Patients. B – Regression coefficients; SE – Standard Error; CI – Confidence Interval; FAS – Fatigue Assessment Scale; 25(OH)D – 25-hydroxyvitamin D; CES-D – Center for Epidemiologic Studies Depression Scale; CXR – Chest X-Ray.

	B	SE	Wald	df	p	Odds<br>ratio	95% CI for the odds ratio		Points
							Lower	Upper	
Serum 25(OH)D ≤20 ng/mL (No) –<br>*Reference*	/	/	/	/	/	/	/	/	0
Serum level of 25(OH) D ≤20 ng/mL (Yes)	0.823	0.393	4.387	1	0.036	2.278	1.054	4.921	+2
Persistent dry cough (No) – *Reference*	/	/	/	/	/	/	/	/	0
Persistent dry cough (No) –	1.101	0.337	10.675	1	0.001	3.006	1.553	5.819	+2
FAS Scores ≥22 (No) – *Reference*	/	/		/	/	/	/	/	0
FAS Scores ≥22 (Yes)	2.030	0.323	39.439	1	0.000	7.611	4.040	14.339	+4
FEV1 predicted <80 % (No) by FVC<br>predicted <80 % (No) – *Reference*	/	/	/	/	/	/	/	/	0
FEV1 predicted <80 % (Yes) by FVC<br>predicted <80 % (Yes)	1.133	0.525	4.663	1	0.031	3.105	1.110	8.683	+2
Diplopia (No) – *Reference*	/	/	/	/	/	/	/	/	0
Diplopia (Yes)	1.484	0.662	5.032	1	0.025	4.411	1.206	16.132	+3
Constant	-3.587	0.448	64.142	1	0.000	.028			

Similarly to the previous regression model (Table 4), omnibus models testing showed that the model coefficients (Table 5) at each subsequent step during the stepwise process were significant and that the estimation of parameters was terminated at iteration number 4 (parameter estimates changed by less than 0.001). The model parameters (from step 1 to step 5) are shown in the following order: 1) (with constant) FAS Scores ≥22 (R^2^=0.231, -2 log likelihood=351.702); 2) Persistent dry cough (R^2^=0.264, -2 log likelihood=341.551); 3) Diplopia (R^2^=0.281, -2 log likelihood=336.180); 4) FEV_1_ <80% predicted by FVC <80% predicted (R^2^=0.294, -2 log likelihood=331.895); 5) Serum 25(OH)D ≤20 ng/mL (R^2^=0.310, -2 log likelihood=327.022).

This model showed good construct validity (Nagelkerke R^2^=0.310), excellent adequacy by the Hosmer-Lemesh test (Chi-square=3.226; p=0.665), and excellent discriminant characteristics (c-statistic = 0.800 with 95% CI from 0.759 to 0.840, p <0.0001). The cut-off points of the DSRISP 2 score were above 4 (with a 95% CI from 2 to 4) for classifying an individual patient in the group with a CES-D score ≥20. Other ROC parameters of the DSRISP 2 score with a 95% CI were: sensitivity = 83.7% (74.2%–90.8%), specificity = 69.75% (64.3%–75.1%), positive predictive value = 43.1% (35.5–55.0%) and negative predictive value = 94.0% (90.1%–96.7%).

## Discussion

In the present study, we showed that a high number (32% and 21.5%, respectively) of sarcoidosis patients have DS, as demonstrated by the CES-D with two cut-off points (16 and 20, respectively).

Vitamin D, which has antioxidant properties and activity in brain tissue, is important for mood disorders prevention or treatments, but serum levels must be followed [24]. Until now, most randomized clinical trials investigating the effects of vitamin D supplementation on mood in healthy persons may not have reached sufficient and well-timed increases in serum 25(OH)D to induce benefits on mood [25].

Eyles et al. [26] showed that the distribution of the 1,25-dihydroxyvitamin D_3_ receptor and 1α-hydroxylase, the enzyme responsible for forming the active vitamin in the human brain (in neurons and glial cells) and that both are particularly widespread in the hypothalamus and dopaminergic neurons of the substantia nigra, while the nucleus basalis of Meynert and the Purkinje cells in the cerebellum expressed 1α-hydroxylase in the absence of the 1,25-dihydroxyvitamin D_3_ receptor. In our previous research in patients with sarcoidosis, we demonstrated that DS was associated with the chronic course of the disease, FAS score ≥22, corticosteroid therapy and hyperechogenic zones in the rostral midbrain, especially in the nucleus ruber, which receives many inputs from the cerebellum [27]. Considering the above, we suggest vitamin D_3_ therapy may significantly eliminate DS in sarcoidosis patients. It is difficult to transmit inputs from the midbrain (due to the absence of 1,25-dihydroxyvitamin D_3_ receptors) to the nucleus rubber, which is anatomically and functionally reduced in sarcoidosis. It has also been proven that the use of Vitamin D_3_ plays a significant role in eliminating DS in patients with seasonal affective disorder and other mood disorders [28]. Vitamin D_3_ therapy may be beneficial in patients with major depression, bipolar disorder, or seasonal affective disorder with high inflammatory biomarkers because inflammation can produce DS in vulnerable individuals by lowering plasma tryptophan and diminishing brain serotonin activity. Also, Vitamin D_3_ therapy may improve the efficacy of selective serotonin reuptake inhibitors in these patients.

We demonstrated that female gender and deficiency of 25(OH)D represent independent risk factors for DS in sarcoidosis patients, with the contribution of +1 and +2 points on the DSRISP 1 score, respectively. However, when these listed risk factors are present together in a sarcoidosis patient (total DSRISP 1 score = 3), without other risk factors from our first multivariate regression model (Table 4), the patient is then classified with a probability of 75% into the group without DS (CES-D <16) and indeed has a CES-D score below 16. This is because the cut-off point for DSRISP 1 is 5, with a classification specificity of 75.4% (95% CI 69.8%–80.4%). Accordingly, we suggest that in sarcoidosis patients with a serum 25(OH)D <20 ng/mL, who additionally have high fatigue or other risk factors (DSRISP score >5), vitamin D_3_ therapy should be started or intensified until the value of 25(OH)D is reached >20 ng/mL with remeasurement of CES-D. This is especially important in cases of lack of medical resources and the possibility of conducting a timely psychiatric examination with the consideration of antidepressant therapy. We emphasize that in the health care system of the Republic of Serbia, measuring the serum 25(OH)D and vitamin D_3_ therapy is the patient’s responsibility. Accordingly, additional convincing may be needed tomotivate these patients to purchase vitamin D_3_ preparations. Baughman et al. noticed that up to 50% of sarcoidosis patients, especially postmenopausal women and those taking corticosteroids, show evidence of increased bone fragility, and it was suggested that vitamin D_3_ supplementation may be withheld in sarcoidosis patients with bone fragility unless calcitriol levels are below normal limits [29]. Our results derived by multivariate analysis suggest a different approach may be needed since we found that the serum level of 25(OH)D ≤20 ng/mL represents an independent risk factor from the female gender for DS in sarcoidosis patients (Table 4). Additionally, this is supported by the univariate methods with an even distribution of corticosteroid therapy between groups defined by both (16 or 20) criterion values of CES-D for DS (Table 3).

Univariate analyses showed that extrapulmonary sarcoidosis represents a risk factor for DS in these patients. However, in both our multivariate regression models, this was not confirmed. We previously demonstrated in another sarcoidosis population that patients with extrapulmonary manifestations had more severe symptoms, restrictions of activities of daily living, and impairment of their health status [30].

Fatigue is a major problem in sarcoidosis [31]. Our sarcoidosis patients with DS had more frequent FAS scores ≥22. This follows the results of Mokros et al., who demonstrated that DS were among the significant predictors of FAS scores in sarcoidosis patients [32]. We showed that the presence of both FAS score ≥22 and serum level of 25(OH)D ≤20 ng/mL (DSRISP score = +6) leads to increased DS (Table 4 and Table 5). The high odds ratio (Table 4 and Table 5) associated with FAS Scores ≥22 with the leading partial R^2^ relative to the partial contributions to the R2 of the other predictors in our logistic regression models suggests fatigue’s critical role in DS. Charoenporn and Charernboon demonstrated that the prevalence of vitamin D insufficiency and deficiency was high in patients with long COVID, with female gender as predictors of lower vitamin D levels. However, no associations were found between vitamin D level and fatigue syndrome, depression, anxiety, sleep problems, or cognitive function [33].

It has been proven that fatigue (measured with FAS), after eliminating some socioeconomic confounders in patients with rheumatoid arthritis, plays a significant role in predicting increased DS, which also includes major depression [34].

Although high-resolution computed tomography and FDG-PET provide more information than CXR to help guide treatment decisions, we instead have used chest radiographic staging of pulmonary sarcoidosis according to the Scadding criteria [35] that has been the mainstay of staging thoracic sarcoidosis for decades with fair interobserver concordance [36]. The nodal and parenchymal involvement pattern is typically used to ‘stage’ sarcoidosis according to Scadding criteria into 5 stages. We found that CXR stage 3, which characterizes pulmonary parenchymal disease involvement (without nodal enlargement) and increased fatigue, represent the most substantial risk factors for DS in sarcoidosis patients (Table 4). Therefore, the additive effect ofCXR stage 3 and increased fatigue indicate that a sarcoidosis patient has DS only if CES-D scores ≥16 are used as cut-off points. Since CXR stage 3 was not a predictor of DS in our second multivariate model (Table 5), we believe that CXR stage 3 can generate false-positive cases of DS, applying the criterion value for CES-D of 16. This is supported by the fact that persistent dry cough, increased fatigue, and the serum 25(OH)D <20 ng/mL have the same assigned points in both multivariate regression models (Table 4 and Table 5). Also, when constructing the DSRISP 2 score, cut-off points of CES-D scores ≥20 were used. This resulted in better sensitivity and specificity of the DSRISP 2 score than the DSRISP 1 score.

Cough is a frequent and troublesome symptom of sarcoidosis that reduces patients’ quality of life [20] [37]. We found that persistent dry cough represents an independent risk factor for DS in sarcoidosis patients. Its contribution in both DSRISP scores is +2 (Table 4 and Table 5). We suggest that persistent dry cough in sarcoidosis should be treated with antitussives, mainly when eliminating this risk factor can decrease the DSRISP 1 score below 5, i.e. DSRISP 2 score below 4.

We showed that the chronic course of sarcoidosis, in terms of assigned points (+2), contributes equally to DS as serum 25(OH)D <20 ng/mL or the presence of persistent dry cough (Table 4).

Although many of our patients had DS and/or fatigue, all had normal pulmonary function. All spirometry parameters and DLCO were in normal ranges. This confirms previous studies demonstrating that pulmonary function testing cannot function as a surrogate for other parameters and cannot be used to assess the overall health of sarcoidosis patients [38]. We noticed that DLCO was significantly lower in the group of patients with CES-D score ≥16, and the same trend has been seen in the study of de Vries et al., whose sarcoidosis patients with FAS ≥22 had significantly lesser DLCO values in comparison with those without fatigue [31].

In our study, we demonstrated that the simultaneous presence of FEV_1_ <80% predicted and FVC <80% predicted is a significant predictor of CES-D scores ≥20 with a share of +2 points in the DSRISP 2 score (Table 5). Such spirometric findings (without decreased FEV_1_/FVC) denote pulmonary restriction [39] [40], and this may indicate that due to DS, these patients felt weaker and could not fully cooperate with the spirometric procedures.

We showed that diplopia was a significant predictor of DS in the CES-D ≥20 patients’ group, with +3 points assigned in DSRISP 2 scores (Table V). Sève et al. indicate a high prevalence of ocular involvement in sarcoidosis patients, ranging from 10% to 50% [41]. However, reports of diplopia as an independent predictor of DS in sarcoidosis patients are lacking before our study.

One limitation of this study is that the data were collected in a tertiary healthcare setting. Based on the clinical and radiographic criteria, our patients had predominantly severe forms of the disease and the DS and other subjective outcomes scores were probably more severe than the average sarcoidosis patient. Therefore, our findings may not reflect the greater population of patients treated in primary healthcare institutions, where there are less severe patients regarding their CXR stage and level of fatigue. In addition, one-third of our patients were receiving corticosteroids, and this may have had a negative impact on their DS (their classic adverse effect). Corticosteroid therapy can potentially be a confounding factor for DS in patients with sarcoidosis, as it can stand in the way from cause to effect, i.e. from the level of sarcoidosis activity (high degree of inflammation associated with the inclusion of corticosteroids) to the effect (high CXR, high degree of lung restriction). Our study did not monitor the degree of inflammation with specific markers characteristic of sarcoidosis activity [20] to clarify the confounding effect further.

Seasonal variations in sun exposure can affect vitamin D levels [42]. However, it has been shown that although places closer to the equator have greater sun exposure, it has been reported thatamong residents in these areas, vitamin D insufficiency persists [43]. Our study did not measure the seasonal variation of vitamin D levels in patients with sarcoidosis.

We conclude that serum level of 25-hydroxyvitamin D below 20 ng/mL, as well as persistent dry cough with increased fatigue, represent significant risk factors for DS in sarcoidosis patients with equal predictive influence assessed through both CES-D ≥16 and CES-D ≥20 thresholds criteria. The construction of the Depressive Symptoms Risk Index for Sarcoidosis Patients is recommended exclusively as a procedure for validating multivariate predictive models since this procedure numerically more precisely measures the individual and combined effects of predictors of DS in these patients. We suggest further research with the construction of validated multivariate predictive models to evaluate the possibility of implementing therapy with antitussives alone or with Vitamin D_3_ to decrease the risks of the appearance of DS in sarcoidosis patients.

## Dodatak

### List of abbreviations

CES-D, Center for Epidemiologic Studies – Depression Scale;

CXR, chest X-ray; 

DSRISP, depressive symptoms risk index for sarcoidosis patients; 

FAS, fatigue assessment scale; 

FEV_1_, forced expiratory volume in one second; 

FVC, forced vital capacity; 

DS, depressive symptoms; 

25(OH)D, 25-hydroxyvitamin D.

### Conflict of interest statement

All the authors declare that they have no conflict of interest in this work.
